# Effects of Interactivity on Recall of Health Information: Experimental Study

**DOI:** 10.2196/14783

**Published:** 2020-10-28

**Authors:** Emília Margit Pajor, Sander Matthijs Eggers, Hein de Vries, Anke Oenema

**Affiliations:** 1 Department of Health Promotion, Care and Public Health Research Institute Faculty of Health Medicine and Life Sciences Maastricht University Maastricht Netherlands; 2 International Trade Statistics Netherlands Heerlen Netherlands

**Keywords:** Interactivity, cognitive involvement, active control, cognitive load, recall, need for cognition, health literacy, online health information, information processing, dietary supplements

## Abstract

**Background:**

Information provided in an interactive way is believed to be engaging because users can actively explore the information. Yet empirical findings often contradict this assumption. Consequently, there is still little known about whether and how interactivity affects communication outcomes such as recall.

**Objective:**

The aim of this study was to investigate mechanisms through which interactivity affects recall of online health information. We tested whether and how cognitive involvement, perceived active control, and cognitive load mediate the effects of interactivity on recall. In addition, we examined need for cognition and health literacy as potential moderators of the mediation effects. Given the increasing popularity of dietary supplement use, our health website focused on this topic.

**Methods:**

In an online between-subjects experiment (n=983), participants were randomly assigned to control condition (no interactive features), moderate interactivity (dropdown menus), and high interactivity (dropdown menus and responsive infographics). Two weeks before the experiment, background characteristics and moderating variables were measured. During website visit, data on users’ online behavior were collected. Recall was measured postexposure.

**Results:**

Participants recalled significantly less information in the moderate (mean 3.48 [SD 2.71]) and high (mean 3.52 [SD 2.64]) interactivity conditions compared with the control condition (mean 5.63 [SD 2.18]). In the mediation analysis, we found direct, negative effects of moderate (b=–2.25, 95% CI –2.59 to –1.90) and high (b=–2.16, 95% CI –2.51 to –1.81) levels of interactivity on recall as well. In the relationship between interactivity and recall, cognitive involvement had a partial negative mediation effect (moderate interactivity: b=–.20; 95% CI –0.31 to –0.10; high interactivity: b=–.21, 95% CI –0.33 to –0.10) and perceived active control had a partial positive mediation effect (moderate interactivity: b=.28, 95% CI 0.18 to 0.40; high interactivity: b=.27, 95% CI 0.16 to 0.40).

**Conclusions:**

Interactivity decreased recall. In addition, through interactivity participants were less involved with the content of the information, yet they felt they had more control over the information. These effects were stronger in the high need for cognition and high health literate groups compared with their counterparts.

## Introduction

### Relevance and Aim

One of the unique features of internet-delivered health information is that it can be provided in an interactive way. Interactive features are thought to create more engagement and involvement with the information, as visitors can actively interact with the information [1]. However, clear guidelines are lacking on how interactivity could be used in health communication [2]. Moreover, previous research has resulted in inconclusive findings regarding how interactive features affect communication outcomes. Interactive features may enhance user enjoyment, positive attitudes, and desirable behavioral intentions but they do not necessarily improve cognitive elaboration or information recall [3]. The aim of this study is to investigate whether and how interactivity can be used for improving recall of online health information. Given the increasing popularity of dietary supplement use and the complexity of the behavior [4,5], our study focuses on this topic.

### Theoretical Background

#### Conceptualization of Interactivity

In interactivity research, three approaches are distinguished: structural, experiential, and message exchange. In the structural approach, interactivity is conceptualized in terms of the technical attributes of the medium [6]. Such technical attributes include on-screen interactive features such as menus that allow user-to-system or user-to-user interactions [7]. According to the experiential approach, interactivity is the user’s subjective perception of the level of the medium’s interactivity [8]. The message exchange approach regards interactivity as an ongoing communication process in which (semantic) meanings between two or more communicators are exchanged [9]. We conceptualize interactivity according to the structural approach, since in terms of causality a media stimulus that is manipulated precedes users’ responses to that stimulus [6]. Yet the structural approach often has been criticized for focusing only on direct relations between ae media stimulus and the dependent measures omitting the possibility of third variable effects [6]. Therefore, in the conceptual model of this study, four types of variables were included: interactivity (manipulated independent variable), possible mediators, possible moderators, and a dependent variable.

#### Moderated Mediation Model of Interactivity Effects

In our conceptual model, we will test whether cognitive involvement, perceived active control, and cognitive load mediate the relationship between interactivity and recall. In addition, we will look at whether the need for cognition and health literacy moderate the proposed mediation effects.

Dual process models, such as the elaboration likelihood model (ELM), propose that information elaborated via the central route is likely to produce greater and more permanent changes in communication outcomes [10]. Individuals tend to get (more) motivated to process the information systematically, which involves effortful thinking, if the information is perceived as personally relevant to them [10]. It is proposed that individuals generate (more) cognitive responses (ie, the number of content-related thoughts generated during exposure to the stimuli) to the information content if they process the information systematically [11]. Interactive environments may stimulate cognitive responses since they enable nonlinear, cognitively flexible information use (cognitive flexibility theory) [12]. For instance, navigation through hypertext might be beneficial for knowledge since hypertexts mimic the associative network of human memory [13]. If individuals are afforded the possibility to adapt their information use to their own preferences and cognitive needs, they may get more involved and more motivated to process the information more deeply and elaborate better on the content [14,15]. Therefore, we propose that interactivity improves recall through higher levels of cognitive involvement.

Research has shown that individuals’ perceptions about interactivity are closely related to communicational outcomes such as attitudes [16,17]. From the different dimensions of perceived interactivity distinguished by Liu and Shrum [18], the control dimension, which refers to the autonomy individuals have in controlling the information flow, has been most often associated with cognitive elements of information processing [19]. Active control is characterized by voluntary and instrumental actions through which users are able to customize the information flow [18]. This entails navigational choices based on the user’s own goals and wills [18]. We assume that if users are afforded the possibility to make conscious choices about the information flow based on their needs, they might be more motivated to process the information. Therefore, we propose that interactivity may improve recall through higher levels of perceived active control.

Interactivity may challenge individuals’ information processing capacities by putting an extra burden on users. Tremayne and Dunwoody [20] found evidence that when users visited a more (vs less) interactive website, much cognitive effort was spent in navigation and orientation which had a detrimental effect on recall. In interactive environments, individuals must complete different tasks performed concurrently (eg, reading, navigating). Every task generates a cognitive cost on the working memory in terms of cognitive load. Moreover, tasks may interfere with each other since they compete for the same limited cognitive resources [21,22]. Consequently, there are fewer capacities left for information processing (ie, encoding, storage, retrieval). Indeed, research has shown that individuals retain less information when performing more than one task at the same time because multitasking inhibits the transfer of information into the short- and long-term memory [23,24]. Therefore, we assume that cognitive load increases with higher levels of interactivity, which may lead to decreased levels of recall.

#### Moderators: Need for Cognition and Health Literacy

In addition to the mediation effects described above, we aim to explore whether individual difference variables moderate the proposed mechanisms. Information processing is influenced by individuals’ ability and/or motivation to process information [10,22]. Need for cognition reflects the tendency to engage in and enjoy effortful cognitive endeavors [25,26]. It is considered a stable trait that may be influenced by situational factors such as interactivity [27]. Evidence suggests that interactivity improves information processing, especially among low need for cognition individuals [28]. This may be related to the fact that individuals with low need for cognition prefer interactive websites more than their high need for cognition counterparts [29]. In situations with low personal relevance, individuals with low need for cognition are more attracted by peripheral cues which are attributes that are not inherent to the strength of arguments in the message [30]. In contrast, individuals with high need for cognition concentrate more on the real attributes of the information, such as the strength of arguments. Therefore, they rely less on the way information is presented [28]. However, high need for cognition individuals are more cognitively immersed when engaging in interactive websites than low need for cognition individuals [28,31]. According to the findings of Sicilia et al [28], in both low and high need for cognition individuals, the flow experience increases when they visit an interactive (vs noninteractive) website, but this increase is higher among individuals with high need for cognition.

**Figure 1:**
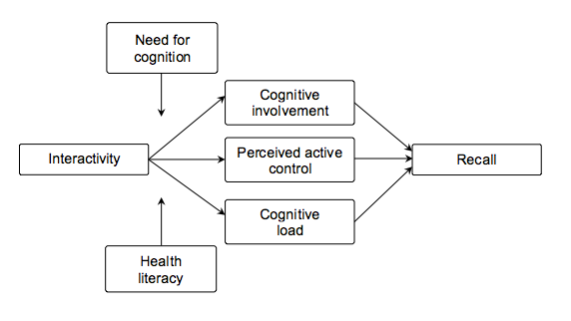
Conceptual model of moderated mediation effects of interactivity.

Individuals may also differ in their ability and skills to understand and use health information. Health literacy entails “the motivation, knowledge, and competencies to access, understand, appraise and apply health information in order to make judgments and take decisions...concerning health care, disease prevention, etc” [32]. In general, low health literate individuals engage less in health information seeking and have greater difficulties with reading and searching for health information on the internet [33,34]. Moreover, interactivity may challenge users with limited literacy skills since they have difficulties with recognizing graphic links (ie, pictures that function as hyperlinks), using navigational tools, and understanding graphics that respond to mouse movements [35]. In addition, sufficient levels of metacognitive skills are needed to make mindful navigational selections and build meaningful sequences of information, for instance, in a hypermedia environment [36]. However, it should be noted that research has shown that interactive features designed specifically for individuals with low health literacy (eg, low text difficulty) are beneficial for online health information processing [37]. We summarized our conceptual model in Figure 1.

In sum, we aim to answer the following research questions: through which mechanisms does interactivity affect recall of health information? Do these mechanisms differ according to individuals’ level of need for cognition and health literacy?

## Methods

### Design

A between-subjects experiment with three levels of interactivity (no interactivity, moderate interactivity, high interactivity) was conducted to investigate the effects of interactivity on cognitive involvement, perceived active control, cognitive load, and recall. Two weeks prior to the experiment, background characteristics and moderator variables were measured. During the experiment, pre- and postexposure measures were performed.

### Participants

**Figure 2:**
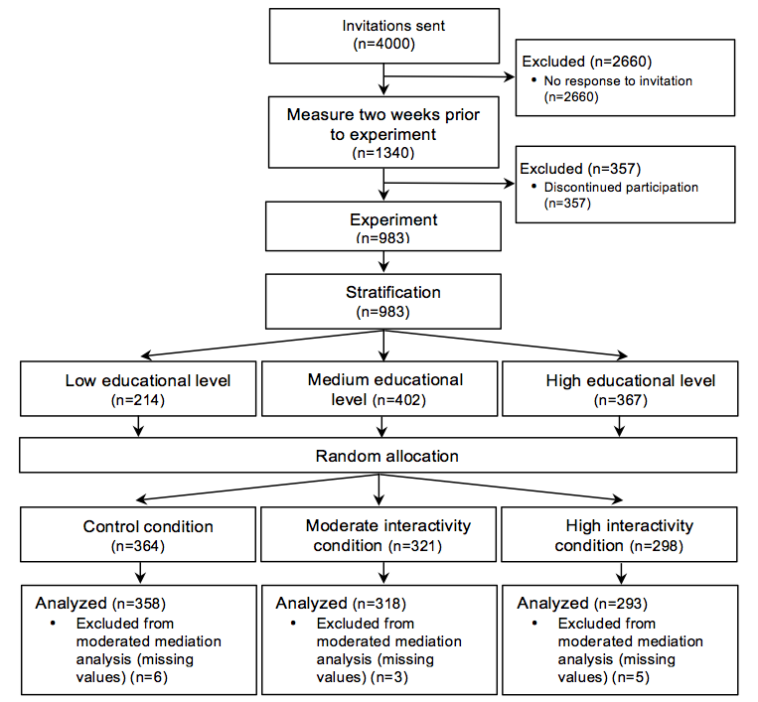
Flowchart of the stratification and random allocation procedure.

A priori power analysis with G*Power indicated that at least 776 participants were needed to detect small effects (ie, *f*^2^=.02) with an alpha level of .05, .90 statistical power, and 4 predictors. Participants were recruited from 26,000 active panel members of I&O Research, an ISO 26362–certified research bureau for access panels in market, opinion, and social research [38]. I&O Research recruits its panel members offline (eg, from municipal registers) and self-registration is not allowed in order to prevent selection biases such as the overrepresentation of frequent internet users [38]. A random sample consisting of 4000 individuals was drawn from the panel, of which 33.50% (1340/4000) responded to the invitation to participate in the first part of the study (see Figure 2). This response rate was comparable to the average response rate of this panel (ie, 35%) [38]. The final sample consisted of 983 (73.4%) individuals who participated in both parts of the study and completed the pre- and postexposure questionnaires. Among these participants, 50 €10 gift cards for an internet warehouse were raffled. Due to technical issues, data of 15 respondents were lost in the preexposure measurement. Therefore, these participants were excluded from the moderated mediation analyses.

### Procedure

An invitation with the subject line “Consumer information on food and dietary supplements” was sent by email to participants on March 21, 2017. The email contained a short description of the study, an explanation of the procedure, and contact details of the researcher. Upon agreeing to participate in the study and giving informed consent, individuals were asked to complete a short questionnaire about health literacy, need for cognition, and educational level. Based on individuals’ educational level, the sample was divided into three strata: low, medium, and high educational level. On April 25, 2017, participants were invited to take part in the actual experiment. Prior to visiting the website, individuals completed a preexposure measurement about their knowledge of the research topic and dietary supplement use. Then, within each stratum, participants were randomly assigned to one of the three versions of a website about vitamin B6 and dietary supplements. The random allocation was programmed by the website developer, Done Digital Kft. In order to make the browsing task similar to a real-life online health information search, participants had the freedom to decide what information and in which order they wanted to explore and no specific instructions or time limits were given. Participants were allowed to view the website only once. In order to prevent any preexposure to the stimulus material, the website was not publicly accessible. Once participants were finished browsing on the website, they were directed to the postmeasurement questionnaire.

### Ethical Approval

According to the decision of the Research Ethics Commission of Maastricht University Medical Centre and Maastricht University (decision number: METC 16-4-268), the Medical Research Involving Human Subjects Act does not apply to this study. At the time of the study, further ethical clearance was not required.

### Stimulus Material

In all three conditions, the information presented was identical and aimed to provide complete information about vitamin B6, its physiological effects, how it relates to food, and the risks and benefits of supplementation with vitamin B6. The goal was to offer information that is well balanced in terms of describing advantages and disadvantages of vitamin B6 and improves individuals’ understanding of whether and to whom supplementation with vitamin B6 may be reasonable. The recommendations of the Ottawa Decision Support Tutorial were used [39].

In addition to the textual information, the website included four educative infographics that contained information about the Recommended Dietary Allowance of vitamin B6 by age category and gender, the vitamin B6 content of different food products, scientific evidence of possible health effects of vitamin B6, and safe and unsafe doses of vitamin B6 dietary supplements. Further details about the content and structure of the website are presented in Multimedia Appendix 1.

### Experimental Stimuli

**Figure 3:**
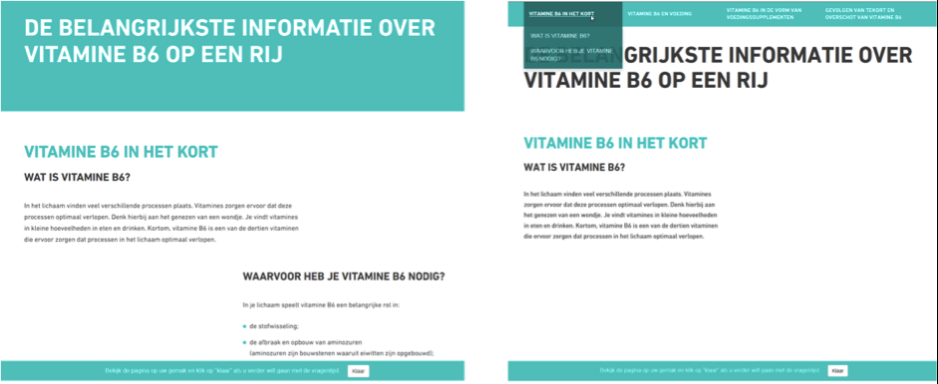
Navigation in the control condition (left) and in the moderate and high interactivity condition (right).

**Figure 4:**
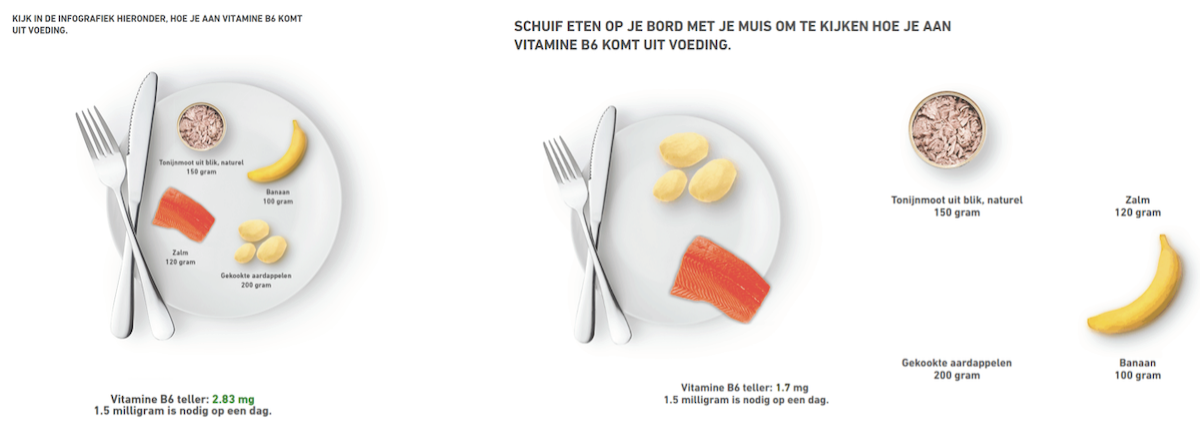
Example of an infographic in the control and moderate interactivity condition (left) and the high interactivity condition (right).

Three versions of a website were developed sharing the same content, layout, and pictures but differing in terms of levels of interactivity. In our study, interactivity refers to the technical attributes of the medium [6]. In line with previous research that falls into the structural approach of interactivity [1,40], we manipulated levels of interactivity in terms of the amount of interactive tools available on the website interface (eg, menus) for accessing and interacting with the content of the website. Accordingly, in the control condition no interactive features were presented. Participants navigated by scrolling up or down on the webpage and the infographics did not respond to users’ mouse movements. In the moderate interactivity condition, a dropdown menu consisting of nine submenus was the only interactive tool presented on the website. The infographics shown in the moderate interactivity condition were static. In the high interactivity condition, two types of interactive features were presented: a dropdown menu and responsive infographics. Three rounds of pilot tests were conducted before the websites were finalized. In Figure 3, the differences in the navigation (scrolling vs dropdown menu) between the conditions are presented. In Figure 4, the differences in the infographics (static vs responsive) between the conditions are presented.

### Measures

#### Dependent Variable: Recall

The measure of recall was based on the construction integration model [41] that distinguishes between two levels of comprehension: the text base level (ie, literal recall of information) and the situation model level (ie, to make inferences to situations based on the information) comprehension. During two cycles of pretesting, 4 questions were developed to measure text base level comprehension and 2 questions to measure situation level comprehension; 3 of the 6 questions were in multiple-choice format in which only one answer option was correct. Three other questions were in multiple response format in which multiple answer options were correct. In both types of response options, the “I don’t know” option was also presented. Participants received 1 point for each correct answer, up to 9 points in total.

According to the principal component analysis, the 6 items loaded on one single underlying construct (eigen value: 2.866, 47.765% of variance explained). In addition, factor loadings were .582 or higher. All 6 items correlated significantly with each other, ranging from *P*=.25 to *P*=.51.

#### Mediating Variables

Participants’ cognitive involvement with the website content was measured with a thought listing task [11]. Individuals were asked to list thoughts about vitamin B6 that came to their mind during their website visit. Participants could list up to 12 thoughts in empty text boxes, each beginning with the statement: “Vitamin B6 is:...” Each field of text that was completed counted as a thought. The cognitive involvement measure was placed second in the postexposure questionnaire, after the 1-item manipulation check of interactivity. Compared with thought listing measures during exposure, obtaining responses after the stimulus can be accomplished without the interruption or distraction from the stimulus [11]. In addition, loss or retention in poststimulus measures of cognitive involvement is negligible [11].

Perceived active control refers to users’ voluntary and instrumental actions that directly influence their website experience [8,18]. Liu’s [8] 4-item measurement of perceived active control was used.

Cognitive load was conceptualized as the perception of “the cognitive capacity that is actually allocated to accommodate the demands imposed by the task” [42]. It was measured with 4 items derived from the Subjective Workload Assessment Technique (mental effort subdimension) [43,44] and from the NASA Task Load Index (effort subdimension) [43,45].

#### Moderating Variables

Need for cognition was measured with 7 items derived from the Dutch version of the 18-item Need for Cognition Scale (NCS) [46]. A limited number of items may be sufficient to measure need for cognition since evidence suggests that the full NCS measures a single underlying construct [26,47,48]. Therefore, the items with the highest factor loadings were chosen from the study of Hevey and colleagues [49].

Health literacy was measured using the Newest Vital Sign in Dutch (NVS-D), which was developed to measure an individual’s capacity to assess, understand, and use textual and numerical health information [50]. Consequently, this 6-item tool tests three types of skills that are important in finding and interpreting health information: math, locate-the-information (by reading and comprehending), and abstract reasoning (ie, making inferences from the information to specific situations) [51]. For each correct answer, respondents received 1 point, up to 6 points in total. Details of the measurement scales (number of items, example items, answer categories, mean scores, and Cronbach α) are presented in Table 1.

**Table 1:** Overview of number and examples of questions, answering categories, mean scores, and Cronbach 𝛼.

Variable	Number of questions	Example of questions/items, answer options	Mean (SD)	Median^a^ (IQR)	Cronbach alpha
Recall^b^	6	“Some groups of individuals are at risk of developing vitamin B6 deficiency. Which groups are these?” “Someone is using high-dose vitamin B6 dietary supplements (100 milligram) for a long period of time. What kind of influence can this have on the health condition of this person?” Multiple choice or multiple response answer options	4.30 (2.71)	—^c^	.77
Cognitive involvement^d^	12	“During your website visit, certain thoughts about vitamin B6 may have come to your mind. Please write down your thoughts about vitamin B6 in the text boxes below.” Open-ended questions	—	3 (3)	—
Perceived active control	3	“While I was on the website, I could choose freely what I wanted to see.” Totally disagree (1) to totally agree (7)	3.98 (1.29)	—	.80
Cognitive load	4	“I had to think hard in order to understand the information on the website” Totally disagree (1) to totally agree (7)	4.05 (1.42)	—	.92
Health literacy^e^	6	“If you are allowed 60 grams of carbohydrates as a snack, how much ice cream could you have?” Open-ended questions	—	6 (1)	—
Need for cognition	7	“I really enjoy a task that involves coming up with new solutions to problems” Totally disagree (1) to totally agree (7)	4.94 (1.02)	—	.83

^a^For variables with non-normal distribution, the median and IQR are presented.

^b^Recall scores could range from 0 (no correct answers) to 9 (all answers are correct).

^c^Not applicable.

^d^Cognitive involvement scores could range from 0 (no thoughts) to 12 (12 thoughts).

^e^Health literacy scores could range from 0 (no correct answers) to 6 (all answers are correct).

### Manipulation Check Measure

The effectiveness of the manipulation was measured by asking respondents: “To what extent do you agree with the following statement: This website is interactive” with answer options ranging from 1 (totally disagree) to 7 (totally agree) [52].

### User Activity

During participants’ website visit, the following user activity indicators were measured: duration of website visit in seconds (mean 11,765.69 [SD 92,169.31], range 5-1,302,883), the extent of scrolling down on the website (control condition: mean 0.98 [SD 0.32]), total amount of clicks on the nine menus (moderate interactivity condition: mean 6.11 [SD 3.45]; high interactivity condition: mean 6.13 [SD 2.64]), and total amount of clicks on the four infographics (high interactivity condition: mean 11.05 [SD 9.09]). Due to personal browser settings such as a disabled JavaScript [53], user activity data of 524 participants out of the 983 participants were collected.

### Control Variables

Several variables were measured to control for their potential influence in the statistical analyses. The following demographic background characteristics were measured: gender, age, and highest educational level. The latter was measured with 7 responses: 1=primary education or less, 2=preparatory secondary vocational education (level 1) or equivalent, 3=secondary vocational education, 4=senior secondary vocational education (level 2-4) or equivalent, 5=senior general secondary education, university preparation education, 6=bachelor’s level or equivalent, and 7=master’s level or above. The strata of low (1-3), middle (4 and 5), and high educational level (6 and 7) were based on these responses. In addition, meat consumption, diet, mode of life (eg, anthroposophic nutrition), dietary supplement use, and involvement with the topic vitamin B6 were measured. Involvement was measured with 3 items from Zaichkowsky’s [54] personal involvement inventory scale (eg, “The topic vitamin B6 is important to me,” 1=totally disagree to 7=totally agree).

### Statistical Analyses

Descriptive statistics were run to investigate sample characteristics. In order to check normal distribution of the variables, we looked at skewness and kurtosis and visually inspected the histogram and boxplot of each variable. We detected nonnormal distribution in three variables: health literacy (skewness: –1.67, kurtosis: 2.28), cognitive involvement (skewness: 1.48, kurtosis: 3.68), and time spent on website (skewness: 9.42, kurtosis: 101.41). To investigate the proposed mediations and examine whether differences exist between subgroups, the PROCESS macro for SPSS Statistics version 2.16.3 (IBM Corporation) was used [55]. PROCESS applies bootstrapping to estimate 95% bias-corrected confidence intervals for total and indirect effects. Bootstrap procedures are unaffected by violations of parametric assumptions and have higher type I error control and power than the normal theory approach [55,56]. In PROCESS, 76 different conceptual diagrams are available. Model number 4 is programmed to test a simple mediation. In order to test mediations, model 4 (10,000 samples) was used with three mediators operating in parallel: cognitive involvement, perceived active control, and cognitive load. In the analyses, the independent variable interactivity was defined as multicategorical; consequently, it was automatically dummy coded by PROCESS (D1: moderate interactivity condition; D2: high interactivity condition; reference category: control condition). The percentage mediated effect was calculated for each significant indirect effect separately by dividing the corresponding unstandardized regression coefficient of the ab path (indirect effect) by the unstandardized regression coefficient of the c path (total effect) and multiplying it by 100. To examine differences between subgroups regarding the mediations (high vs low health literacy, high vs low need for cognition), model 4 (10,000 samples) was run for each group separately. Subgroups were created based on a mean split. This approach of testing moderated mediation effects was chosen for a technical reason. PROCESS v2 had limited features for dealing with multicategorical variables, which meant that it was not possible to calculate an interaction term of a multicategorical and a numerical variable and put it in the model as a variable [57]. All analyses were conducted with adjustments for gender, age, educational level, dietary supplement use, involvement with the topic vitamin B6, being on a diet, following a certain rule of life (eg, anthroposophy), meat consumption, and duration of website visit. Analyses were conducted with SPSS version 23.

## Results

### Sample Characteristics

Slightly more female (530/983, 53.9%) than male (453/983, 46.1%) individuals participated in the study. Participants were on average aged 53.2 (SD 15.31) years, and most of them held either a medium (402/983, 40.9%) or high educational level (367/983, 37.3%; Table 2). More than half of the sample (524/983, 53.3%) had used dietary supplements in the last 12 months. Participants can be regarded as neutral toward the topic vitamin B6 since they were moderately involved (mean 3.42 [SD 1.54]).

**Table 2:** Sample characteristics (n=983).

Characteristic		Value
Gender (male), n (%)		453 (46.1)
Age in years, mean (SD)		53.20 (15.31)
**Educational level, n (%)**		
	Low	214 (21.8)
	Medium	402 (40.9)
	High	367 (37.3)
Living according to a specific mode of life (yes), n (%)		92 (9.4)
Meat consumption (yes), n (%)		945 (96.1)
Being on a diet (yes), n (%)		201 (20.4)
Dietary supplement use in the last 12 months (yes), n (%)		524 (53.3)
Involvement with the topic vitamin B6^a^, mean (SD)		3.42 (1.54)

^a^Involvement was measured on a 7-point Likert scale. The higher the score, the more involved participants were.

### Manipulation Check of Interactivity

The analysis of variance revealed significant differences between the three versions of the website regarding interactivity (*F*_2,980_=24.99, *P*<.001, ηp^2^=.05). Post hoc Bonferroni tests indicated that all three versions differed significantly from each other. Participants rated the level of interactivity as low in the control condition (mean 3.31, SE .10, n=364), as moderate in the moderate interactivity condition (mean 3.89, SE .10, n=321), and as high in the high interactivity condition (mean 4.30, SE .10, n=298). Thus, the manipulation was successful.

### Descriptive Statistics of User Actions Within Conditions

Users spent the most time (minutes) browsing the website in the control condition (median 2.62 [IQR 1.54-3.96]), followed by the moderate (median 0.8 [IQR 0.45-2.84]) and high interactivity condition (median 0.83 [IQR 0.43-3.23]). In the control condition, 78.7% (203/258) of participants scrolled all the way down the website and viewed all website content. In the moderate interactivity condition, 14.8% (21/142) of users clicked on all dropdown menus and viewed the complete content, whereas in the high interactivity condition only 8.9% (11/124) did so. Within the high interactivity condition, the proportion of participants who used all infographics was higher (36/124, 29.0%) than of those who used all dropdown menus (11/124, 8.9%). Descriptive statistics of user actions within conditions are presented in Table 3.

**Table 3:** Indicators of user activity per condition (n=524).

Variable	Control condition (n=258)	Moderate interactivity condition (n=142)	High interactivity condition (n=124)
Duration of website visit in minutes, mean (SD) [range]	366.19 (2146.69) [0.08-21714.72]	112.54 (1103.02) [0.08-13083.52]	78.05 (893.48) [0.10-11599.07]
Scrolled to end of browser window^a^, % (modus) [median]	78.7 (1) [1]	N/A^b^	N/A
Used all dropdown menus^c^, % (modus) [median]	N/A	14.8 (7) [6]	8.9 (7) [6]
Used all infographics^d^, % (modus) [median]	N/A	N/A	29.2 (4) [2]

^a^Scrolled scale ranges from 0 (no scroll down) to 1 (scrolled to end of window at least once).

^b^Not applicable.

^c^Dropdown menu use scale ranges from 0 (no clicks on menus) to 9 (all menus were used at least once).

^d^Infographic use scale ranges from 0 (none of the infographics were used) to 4 (all infographics were used at least once).

### Main Effect of Interactivity on Recall

Analysis of variance showed that recall score differed significantly between the conditions (*F*_2,980_=82.329, *P*<.001, ηp^2^=.144). According to the Bonferroni post hoc test, participants recalled significantly more information in the control condition (mean 5.63 [SD 2.18]) compared with the moderate (mean 3.48 [SD 2.71]) and high (mean 3.52 [SD 2.64]) interactivity conditions.

### Mediating Effect of Cognitive Involvement, Perceived Active Control, and Cognitive Load

**Figure 5:**
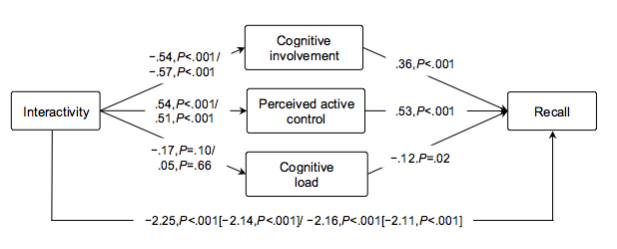
Unstandardized path coefficients of the direct and total effects (in brackets) of moderate and high interactivity on the mediators and recall compared with the control condition (n=968).

Results showed that the effects of levels of interactivity were mediated by cognitive involvement (moderate interactivity: b=–.20, 95% CI –0.31 to –0.10, 9% mediated effect; high interactivity: b=–.21, 95% CI –0.33 to –0.10, 10% mediated effect) and perceived active control (moderate interactivity: b=.28, 95% CI 0.18 to 0.40, 13% mediated effect; high interactivity: b=.27, 95% CI 0.16 to 0.40, 13% mediated effect) but not by cognitive load (moderate interactivity: b=.02, 95% CI 0 to 0.07; high interactivity: b=–.01, 95% CI –0.04 to 0.02). The mediations were partial as there was a remaining significant direct effect of interactivity on recall (moderate interactivity: b=–2.25, 95% CI –2.59 to –1.90; high interactivity: b=–2.16, 95% CI –2.51 to –1.81). Levels of interactivity and the three mediators explained 34% of the variance in recall (*F*_14,953_=35.76, *P*<.001). The unstandardized path coefficients of the direct and total effects are presented in Figure 5.

### Conditional Indirect Effect of Interactivity on Recall According to Two Levels of Need for Cognition

The study sample was split into high (ie, score ≥4.95, 519/968) versus low (ie, score ≤4.94, 449/968) need for cognition using a mean split. Results showed that in individuals with low need for cognition exposed to the moderate interactivity condition, the effects of levels of interactivity were mediated by cognitive involvement (b=–.18, 95% CI –0.36 to –0.05, 8% mediated effect) and perceived active control (b=.14, 95% CI 0.01 to 0.30, 6% mediated effect) but not by cognitive load (b=.12, 95% CI –0.03 to 0.08). In low need for cognition individuals exposed to the high interactivity condition, none of the proposed mediations were significant.

**Figure 6:**
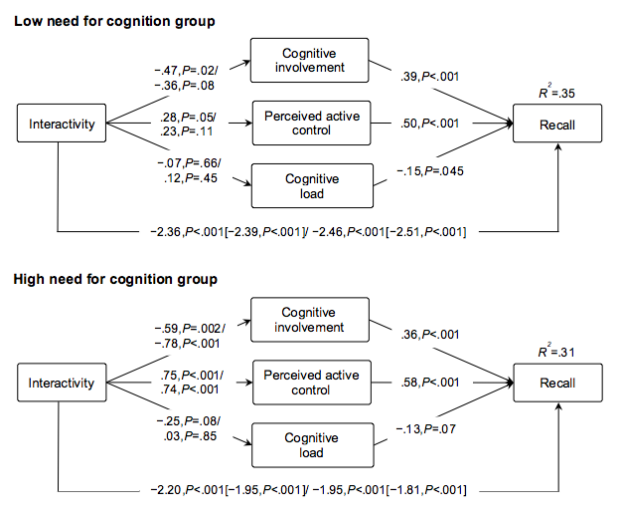
Unstandardized path coefficients of the direct and total effects (in brackets) of moderate and high interactivity on the mediators and recall compared with the control condition in the low (n=449) and high (n=519) need for cognition group.

In individuals with high need for cognition, in the moderate interactive condition a significant mediation effect of cognitive involvement (b=–.21, 95% CI –0.38 to –0.08, 11% mediated effect) and active control (b=.43, 95% CI 0.27 to 0.64, 22% mediated effect) was found. The mediation effects of cognitive involvement (b=–.28, 95% CI –0.46 to –0.14, 16% mediated effect) and perceived active control (b=.43, 95% CI 0.25 to 0.65, 4% mediated effect) were also significant in the high interactivity condition. Regardless of the condition, no mediation effect of cognitive load was found in high need for cognition individuals. As presented in Figure 6, levels of interactivity had a significant direct effect on recall.

### Conditional Indirect Effect of Functional Interactivity on Recall According to Two Levels of Health Literacy

In order to test health literacy as a potential moderator of the mediation effects, individuals were categorized as having a low (ie, score ≤5.14, 379/968 or high (ie, score≥5.15, 589/968) health literacy level using a mean split. Results showed that in low health literate individuals, interactivity effects on recall were mediated significantly only by cognitive involvement (b=–.18, 95% CI –0.37 to –0.03, 7% mediated effect) in the moderate interactivity condition. When levels of interactivity were high, no mediation effects were found in low health literate individuals.

**Figure 7:**
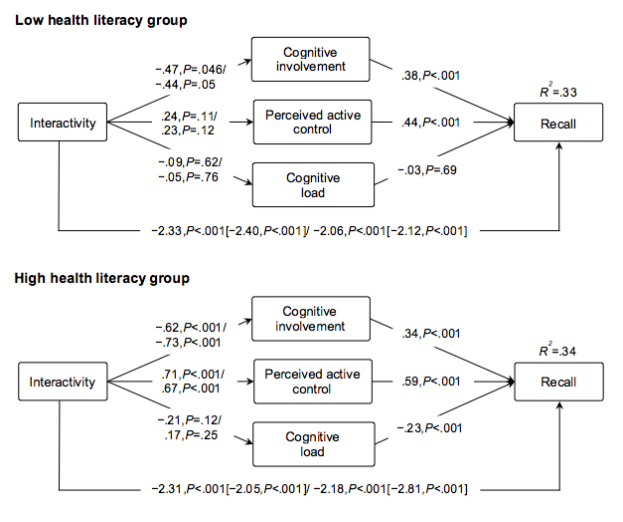
Unstandardized path coefficients of the direct and total effects (in brackets) of moderate and high interactivity on the mediators and recall compared with the control condition in the low (n=379) and high (n=589) health literacy group.

In high health literate individuals exposed to the moderate interactivity condition, interactivity effects were mediated by cognitive involvement (b=–.21, 95% CI –0.36 to –0.09, 10% mediated effect) and perceived active control (b=.42, 95% CI 0.28 to 0.60, 18% mediated effect). In the high interactivity condition, cognitive involvement (b=–.25, 95% CI –0.41 to –0.12, 11% mediated effect) and perceived active control (b=.40, 95% CI 0.22 to 0.61, 20% mediated effect) also partially mediated the effect. Direct and total effects are presented in Figure 7.

## Discussion

### Principal Findings

Our study aimed to examine whether and how cognitive involvement, perceived active control, and cognitive load influenced the relationship between moderate and high levels of interactivity and recall. In addition, we looked at whether those pathways differed according to individuals’ level of need for cognition and health literacy. Two out of the three mediation effects we tested were significant: a negative indirect effect of interactivity on recall was found through cognitive involvement and a positive indirect effect was found through active control. Cognitive load did not mediate the relation between interactivity and recall. In addition, the mediations found were partial since beside indirect effects, interactivity also had direct negative effects on recall. Participants in the moderate and high interactivity conditions remembered less information compared with the control condition.

With regard to cognitive involvement, we found a small, negative indirect effect that indicates that moderate and high levels of interactivity reduced recall through reduced cognitive involvement (ie, fewer thoughts generated). According to the ELM, individuals’ cognitive responses such as the number of thoughts generated increase if information processing follows the central route [58]. Our results may indicate that higher levels of interactivity hinder systematic information processing, resulting in less recall. This assumption is in line with previous research in which participants generated significantly fewer thoughts when exposed to interactive features (vs noninteractive features) [59]. A possible explanation for these findings might be that when individuals are exposed to interactive media that require different types of user actions (eg, reading, navigating), individuals may split their cognitive resources between the tasks. Since information processing capacities are already limited, this may lead to less conscious thinking about the message content [22,60]. Jeong and Hwang [61] found that media multitasking hindered systematic information processing resulting in reduced levels of attention, comprehension, and recall.

Our results showed that moderate and high levels of interactivity improved recall indirectly through enhanced perceptions of active control. In terms of dual process models, this may provide additional evidence that users tend to take the peripheral route of information processing when using interactive media. Sundar and Limperos [62] argue that new media offers several types of technical affordances (eg, navigability) that may serve as cues (ie, snap judgments). For instance, the browsing heuristic refers to users’ online information-seeking behavior in which they skim the site content and check out the menus or hyperlinks superficially [63]. Indeed, our data on user activity showed that participants did not make use of all interactive functions of the moderately and highly interactive websites. However, the magnitude of the mediation effect of perceived active control was comparable in both experimental conditions. As previously suggested, perceived active control may not be a function of the quantity but of the type of interactive features presented on a website [64].

Since we found no mediation effect of cognitive load, we suppose that moderate and high levels of interactivity are not more cognitively demanding in terms of information processing than static content (ie, control condition). Website complexity is a function of page length; amount of information presented; and number of pictures, hyperlinks, or other elements embedded in the website [65]. Our nonsignificant finding might be explained by the fact that we did not vary website content across conditions; we only varied the amount of interactive tools through which participants could interact with the website.

The direct, negative effect of interactivity on recall might be explained by user activity data revealing that both time spent on the website and amount of content visited were the highest in the control condition. This may imply that if users are provided with interactive features, their information search becomes more purposive, and the increased selectivity exposes them to less information, resulting in less recall.

It should be noted that in our study we examined the effects of cognitive involvement, perceived active control, and cognitive load in three separate pathways between interactivity and recall. However, these concepts should be examined in relation to each other as well. For instance, users’ sense of high active control might be related to cognitive involvement. Active control entails autonomous user actions that might be driven by intrinsic interest, which is positively related to focused attention [31]. Therefore, future research should examine whether and how these concepts are related to each other.

### Differences in Mediation Effects With Regard to Need for Cognition and Health Literacy

The partial negative mediation effect of cognitive involvement and partial positive mediation effect of perceived active control were of greater magnitude in individuals with high (vs low) need for cognition and in individuals with high (vs low) health literacy. While need for cognition and health literacy are generally associated with higher levels of elaboration and recall of (health) information [27,66,67], such associations were not found in research on interactive media [28,68], or negative associations were found [69]. Since literature suggests that interactive websites are preferred more by individuals with low need for cognition than their counterparts [29], we assume that interactivity distracts high need for cognition individuals from systematic information processing.

### Limitations

Our study had some limitations. First, we might have found less contrast between low and high health literacy groups since 87.2% of the sample (844/968) had adequate health literacy, according to the categorization of the newest vital sign [50,51]. Second, we did not measure recall of interactive and noninteractive website content separately. Accordingly, conclusions can be drawn only about individuals’ overall recall of the website content. Third, this study was an online experiment that could be completed on participants’ own device at home. Therefore, it is possible that not all participants paid full attention to the website as some outliers were found in the variable duration of website visit. However, analyses yielded comparable results when excluding those 66 outliers from the sample. Fifth, the topic of the study may seem very specific to some participants and appeal only to a selective group of individuals, namely those interested in nutrition and dietary supplements.

### Conclusions

Higher levels of interactivity decreased recall through reduced levels of cognitive involvement. At the same time, higher levels of interactivity increased recall through enhanced perceptions of active control. No significant mediation effects of cognitive load were found. In addition, the identified indirect effects were of greater magnitude in individuals with high (vs low) need for cognition and with high (vs low) health literacy. Beside the indirect effects, levels of interactivity decreased recall directly in all analyses.

## Appendix

Multimedia Appendix 1 Content and structure of the website about vitamin B6.

Multimedia Appendix 2 CONSORT-EHEALTH checklist V1.6.1.

## Abbreviations

ELM: elaboration likelihood model
NCS: Need for Cognition Scale
NVS-D: Newest Vital Sign in Dutch

## References

[1] Sundar SS, Johnson AN, McKenna KYA, Postmes T, Reips U-D (2007). Social psychology of interactivity in human-website interaction. Oxford Handbook of Internet Psychology.

[2] Oh J (2017). The effect of interactivity on smokers' intention to quit: a linear or curvilinear relationship?. Comput Human Behav.

[3] Yang F, Shen F (2017). Effects of web interactivity: a meta-analysis. Comm Res.

[4] van Rossum C, Buurma-Rethans E, Vennemann F, Beukers M, Brants HA, De Boer E (2016). The diet of the Dutch: results of the first two years of the Dutch National Food Consumption Survey 2012-2016. National Institute for Public Health and the Environment.

[5] Gahche J, Bailey R, Burt V, Hughes J, Yetley E, Dwyer J, Picciano MF, McDowell M, Sempos C (2011). Dietary supplement use among U.S. adults has increased since NHANES III (1988-1994). NCHS Data Brief.

[6] Bucy EP, Tao C (2007). The mediated moderation model of interactivity. Media Psychol.

[7] Sundar SS, Xu Q, Bellur S, Oh J, Jia H (2010). Modality is the message: interactivity effects on perception and engagement.

[8] Liu Y (2003). Developing a scale to measure the interactivity of websites. J Advert Res.

[9] Rafaeli S, Hawkins RP, Wiemann JM, Pingree S (1988). Interactivity: from new media to communication. Advancing Communication Science: Merging Mass and Interpersonal Processes.

[10] Petty RE, Cacioppo JT, Petty RE, Cacioppo JT (1986). The elaboration likelihood model of persuasion. Communication and Persuasion: Central and Peripheral Routes to Attitude Change.

[11] Cacioppo JT, Petty RE, Merluzzi TV, Glass CR, Genest M (1981). Social psychological procedures for cognitive response assessment: the thought listing technique. Cognitive Assessment.

[12] Jacobson MJ, Spiro RJ (2016). Hypertext learning environments, cognitive flexibility, and the transfer of complex knowledge: an empirical investigation. J Educ Comput Res.

[13] Jonassen DH, McAleese R, Green C (1990). Semantic network elicitation: tools for structuring hypertext. Hypertext State of the Art.

[14] Merrill MD (1980). Learner control in computer based learning. Computers & Education.

[15] Patterson NG (2000). Hypertext and the changing roles of readers. English J.

[16] Tremayne M (2005). Lessons learned from experiments with interactivity on the web. J Interact Advert.

[17] McMillan SJ, Hwang J-S, Lee G (2003). Effects of structural and perceptual factors on attitudes toward the website. JAR.

[18] Liu Y, Shrum LJ (2002). What is interactivity and is it always such a good thing? Implications of definition, person, and situation for the influence of interactivity on advertising effectiveness. J Advert.

[19] Shrum LJ, Lowrey TM, Liu Y, Nabi RL, Oliver MB (2009). Emerging issues in advertising research. The Sage Handbook of Media Processes and Effects.

[20] Tremayne M, Dunwoody S (2016). Interactivity, information processing, and learning on the world wide web. Sci Comm.

[21] Sweller J (1994). Cognitive load theory, learning difficulty, and instructional design. Learning and Instruction.

[22] Lang A (2000). The limited capacity model of mediated message processing. J Commun.

[23] Edwards MB, Gronlund SD (1998). Task interruption and its effects on memory. Memory.

[24] Lee J, Lin L, Robertson T (2011). The impact of media multitasking on learning. Learning Media Technol.

[25] Cacioppo JT, Petty RE (1982). The need for cognition. J Personality Soc Psychol.

[26] Cacioppo JT, Petty RE, Feng Kao C (2010). The efficient assessment of need for cognition. J Personality Assess.

[27] Cacioppo JT, Petty RE, Feinstein JA, Jarvis WBG (1996). Dispositional differences in cognitive motivation: he life and times of individuals varying in need for cognition. Psychol Bull.

[28] Sicilia M, Ruiz S, Munuera JL (2005). Effects of interactivity in a web site: the moderating effect of need for cognition. J Advert.

[29] Amichai-Hamburger Y, Kaynar O, Fine A (2007). The effects of need for cognition on Internet use. Comput Human Behav.

[30] Kaynar O, Amichai-Hamburger Y (2008). The effects of need for cognition on internet use revisited. Comput Human Behav.

[31] Srivastava K, Shukla A, Sharma N (2010). Online flow experiences: the role of need for cognition, self-efficacy, and sensation seeking tendency. Int J Bus Insights Transformat.

[32] Sørensen K, Van den Broucke S, Fullam J, Doyle G, Pelikan J, Slonska Z, Brand H, (HLS-EU) Consortium Health Literacy Project European (2012). Health literacy and public health: a systematic review and integration of definitions and models. BMC Public Health.

[33] Jensen JD, King AJ, Davis LA, Guntzviller LM (2010). Utilization of internet technology by low-income adults: the role of health literacy, health numeracy, and computer assistance. J Aging Health.

[34] Birru MS, Monaco VM, Charles L, Drew H, Njie V, Bierria T, Detlefsen E, Steinman RA (2004). Internet usage by low-literacy adults seeking health information: an observational analysis. J Med Internet Res.

[35] Zarcadoolas C, Blanco M, Boyer JF, Pleasant A (2002). Unweaving the Web: an exploratory study of low-literate adults' navigation skills on the World Wide Web. J Health Commun.

[36] Lawless KA, Brown SW (1997). Multimedia learning environments: issues of learner control and navigation. Instruct Sci.

[37] Meppelink CS, van Weert JCM, Haven CJ, Smit EG (2015). The effectiveness of health animations in audiences with different health literacy levels: an experimental study. J Med Internet Res.

[38] I&O Research. I&O Research Panel 2017.

[39] O'Connor A, Stacey D, Boland L (2015). Ottawa Decision Support Tutorial (ODST). The Ottawa Hospital Research Institute.

[40] Sundar SS, Bellur S, Oh J, Xu Q, Jia H (2013). User experience of on-screen interaction techniques: an experimental investigation of clicking, sliding, zooming, hovering, dragging, and flipping. Human Comput Interact.

[41] Kintsch W (1988). The role of knowledge in discourse comprehension: a construction-integration model. Psychol Rev.

[42] Paas F, Tuovinen JE, Tabbers H, Van Gerven PWM (2003). Cognitive load measurement as a means to advance cognitive load theory. Educat Psychol.

[43] Eggemeier FT, Wilson GF, Damos DL (1991). Performance-based and subjective assessment of workload in multi-task environments. Multiple-Task Performance.

[44] Reid GB, Potter SS, Bressler JR (1989). Subjective workload assessment technique (SWAT): a user's guide. Armstrong Aerospace Medical Research Laboratory, AAMRL-TR-89-023.

[45] Hart SG, Staveland LE, Hancock PA, Meshkati N (1988). Development of NASA-TLX (Task Load Index): results of empirical and theoretical research. Human Mental Workload.

[46] Pieters RGM, Verplanken B, Modde JM (1987). “Neiging tot nadenken”: Samenhang met beredeneerd gedrag [“Need for Cognition”: the connection with reasoned behavior]. Nederlands Tijdschrift voor de Psychologie en haar Grensgebieden [Dutch Journal of Psychology and its Related Fields].

[47] Dornic S, Ekehammar B, Laaksonen T (1991). Tolerance for mental effort: Self-ratings related to perception, performance and personality. Personality and Individual Differences.

[48] Sadowski CJ (1993). An examination of the short need for cognition scale. J Psychol.

[49] Hevey D, Thomas K, Pertl M, Maher L, Craig A, Ni CS (2012). Method effects and the need for cognition scale. Int J Educ Psychol Assess.

[50] Fransen MP, Leenaars KEF, Rowlands G, Weiss BD, Maat HP, Essink-Bot ML (2014). International application of health literacy measures: adaptation and validation of the newest vital sign in The Netherlands. Patient Educ Couns.

[51] Weiss BD, Mays MZ, Martz W, Castro KM, DeWalt DA, Pignone MP, Mockbee J, Hale FA (2005). Quick assessment of literacy in primary care: the newest vital sign. Ann Fam Med.

[52] Sundar SS, Kalyanaraman S, Brown J (2016). Explicating web site interactivity. Commun Res.

[53] Clifton B (2012). Advanced Web Metrics with Google Analytics. 3rd Edition.

[54] Zaichkowsky JL (1994). The personal involvement inventory: reduction, revision, and application to advertising. J Advertis.

[55] Hayes AF (2013). Introduction to Mediation, Moderation, and Conditional Process Analysis: A Regression-Based Approach. 1st Edition.

[56] Russell CJ, Dean MA (2016). To log or not to log: bootstrap as an alternative to the parametric estimation of moderation effects in the presence of skewed dependent variables. Organiz Res Meth.

[57] Hayes A (2017). What's coming in PROCESS v3.0.

[58] Petty RE, Cacioppo JT, Strathman AJ, Priester JR, Brock TC, Green MC (2005). To think or not to think: exploring two routes of persuasion. Persuasion: Psychological Insights and Perspectives. 2nd Edition.

[59] Oh J, Bellur S, Sundar SS (2015). Clicking, assessing, immersing, and sharing: an empirical model of user engagement with interactive media. Commun Res.

[60] Wu LL, Lin JY (2012). The match between information control and motivation in the online context. Psychol Mark.

[61] Jeong S, Hwang Y (2016). Media multitasking effects on cognitive vs. attitudinal outcomes: a meta-analysis. Hum Commun Res.

[62] Sundar SS, Limperos AM (2013). Uses and grats 2.0: new gratifications for new media. J Broadcast Electr Media.

[63] Sundar SS, Metzger MJ, Flanagin AJ (2008). The MAIN model: a heuristic approach to understanding technology effects on credibility. Digital Media, Youth, and Credibility.

[64] Voorveld HAM, Neijens PC, Smit EG (2011). The relation between actual and perceived interactivity. J Advert.

[65] Huang M (2003). Designing website attributes to induce experiential encounters. Comput Human Behav.

[66] McCarthy DM, Waite KR, Curtis LM, Engel KG, Baker DW, Wolf MS (2012). What did the doctor say? Health literacy and recall of medical instructions. Med Care.

[67] Berkman ND, Sheridan SL, Donahue KE, Halpern DJ, Crotty K (2011). Low health literacy and health outcomes: an updated systematic review. Ann Intern Med.

[68] Lustria MLA (2007). Can interactivity make a difference? Effects of interactivity on the comprehension of and attitudes toward online health content. J Am Soc Inf Sci.

[69] Broekhuizen T, Hoffmann A (2012). Interactivity perceptions and online newspaper preference. J Interact Advertis.

