# Supplementary material for: Effects of Interactivity on Recall of Health Information: Experimental Study
Source: J Med Internet Res. 2020 Oct 28;22(10):e14783. doi: 10.2196/14783 (PMC7657723; doi:10.2196/14783)
Supplement: Multimedia Appendix 1 [file jmir_v22i10e14783_app1.docx]

### Multimedia Appendix 1.


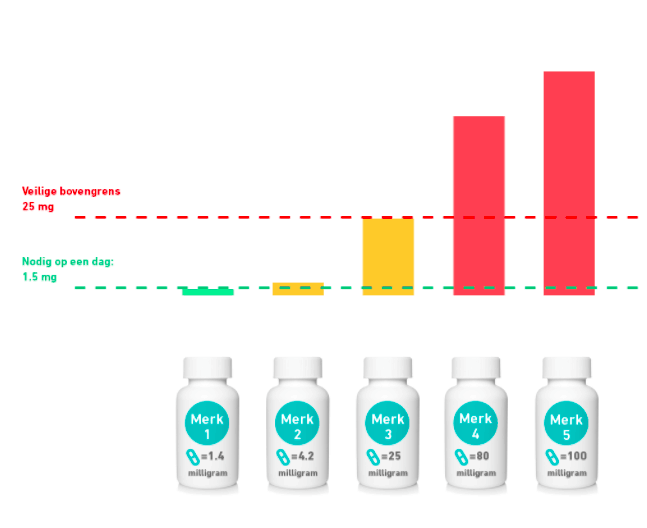

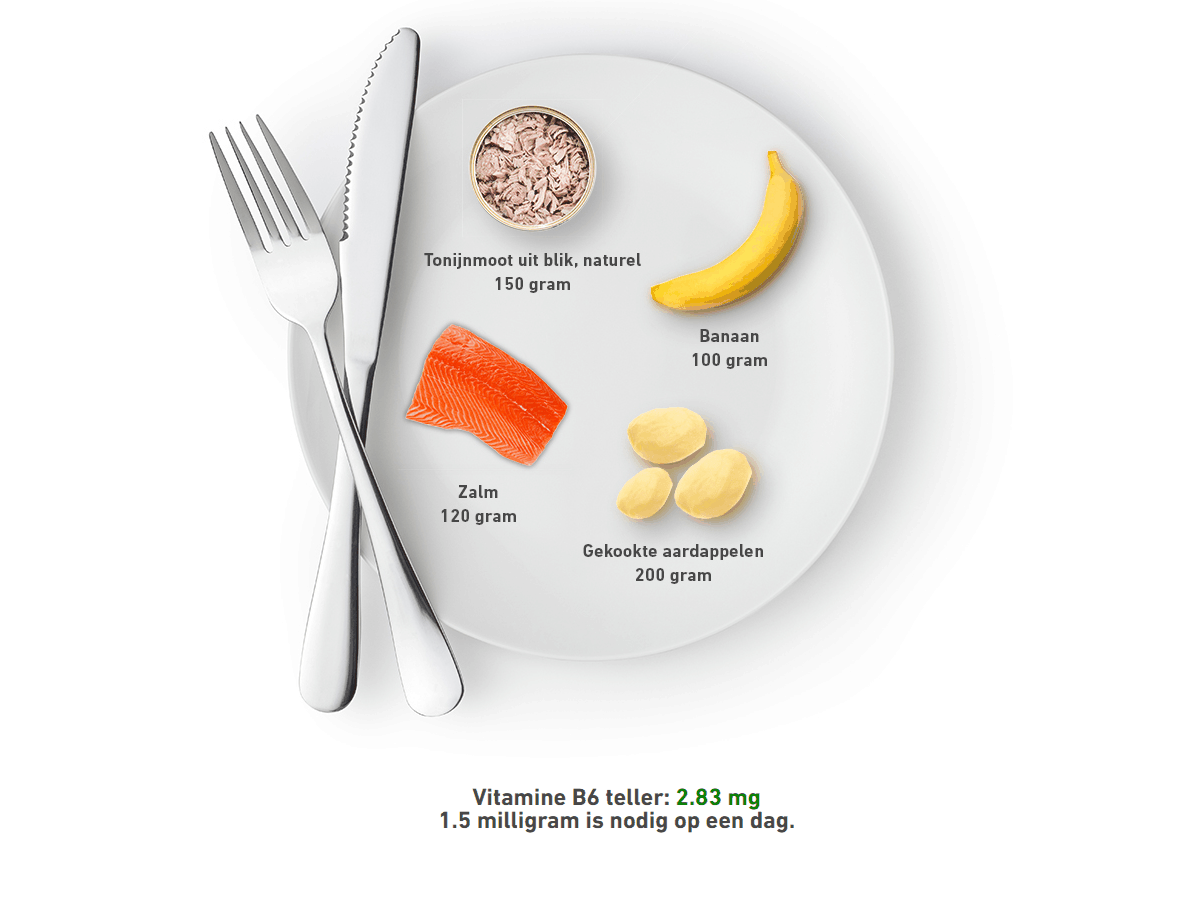

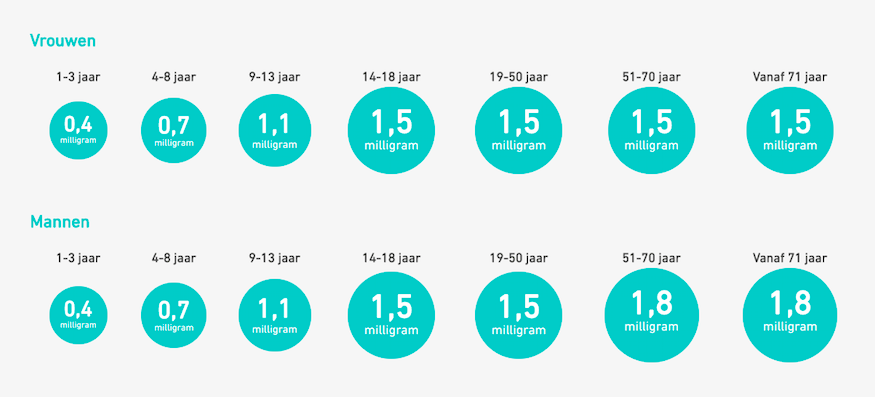
Content and structure of the website about vitamin B6

Infographic nr. 4: Safe and unsafe doses of vitamin B6 dietary supplements

Infographic nr. 3: Scientific evidence of possible health effects of vitamin B6
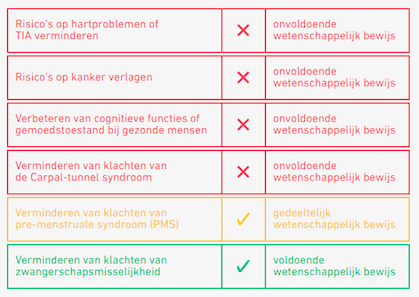


Infographic nr. 2: Vitamin B6 content of banana, canned tuna, boiled potatoes, salmon.

Infographic nr. 1: Recommended dietary allowance (RDA) of vitamin B6 (per age and gender)

What are the consequences of excessive use of vitamin B6 supplements?

What are the consequences of vitamin B6 deficiency?

Consequences of deficiency or excessive intake of vitamin B6

Can you reduce health complaints with vitamin B6?

Vitamin B6 as a dietary supplement

Do you need vitamin B6 supplementation?

How can you get vitamin B6 from food?

What are dietary supplements?

How much vitamin B6 do you need?

Vitamin B6 and diet

What do you need vitamin B6 for?

What is vitamin B6?

Vitamin B6 shortly explained
