# Supplementary material for: Effects of Interactivity on Recall of Health Information: Experimental Study
Source: J Med Internet Res. 2020 Oct 28;22(10):e14783. doi: 10.2196/14783 (PMC7657723; doi:10.2196/14783)
Supplement: Multimedia Appendix 2 [file jmir_v22i10e14783_app2.pdf]

CONSORT-EHEALTH Checklist V1.6.2 Report	Manuscript Number	14783
**(based on CONSORT-EHEALTH V1.6), available at [http://tinyurl.com/consort-ehealth-v1-6].**		
**Date completed**		
10/22/2020 10:33:48		
**by**		
Anke Oenema		
Effects of Interactivity on Recall of Health Information: Experimental Study		
TITLE		
**1a-i) Identify the mode of delivery in the title**		
The study addresses the role of interactivity on recall of information.		
**1a-ii) Non-web-based components or important co-interventions in title**		
**1a-iii) Primary condition or target group in the title**		
Subitem 1a-iii is not applicable. We did not intend to target specific groups in the Dutch population, neither did we intend to tackle specific health-conditions, such as diabetes.		
ABSTRACT		
**1b-i) Key features/functionalities/components of the intervention and comparator in the METHODS section of the ABSTRACT**		
Abstract (methods): "In an online between-subjects experiment (n=983), participants were randomly assigned to control condition (no interactive features), moderate interactivity (dropdown menus), and high interactivity (dropdown menus and responsive infographics)."		
**1b-ii) Level of human involvement in the METHODS section of the ABSTRACT**		
**1b-iii) Open vs. closed, web-based (self-assessment) vs. face-to-face assessments in the METHODS section of the ABSTRACT**		
**1b-iv) RESULTS section in abstract must contain use data**		
**1b-v) CONCLUSIONS/DISCUSSION in abstract for negative trials**		
INTRODUCTION		
**2a-i) Problem and the type of system/solution**		
"Interactive features are thought to create more engagement and involvement with the information, as visitors can actively interact with the information. However, clear guidelines are lacking on how interactivity could be used in health communication."; "The aim of this study is to investigate whether and how interactivity can be used for improving recall of online health information."		
**2a-ii) Scientific background, rationale: What is known about the (type of) system**		
"Interactive environments may stimulate cognitive responses since they enable nonlinear, cognitively flexible information use (cognitive flexibility theory)."; "Interactivity may challenge individuals' information processing capacities by putting an extra burden on users."; " Evidence suggests that interactivity improves information processing, especially among low need for cognition individuals."		
**Does your paper address CONSORT subitem 2b?**		
"The aim of this study is to investigate whether and how interactivity can be used for improving recall of online health information."; " In our conceptual model, we will test whether cognitive involvement, perceived active control, and cognitive load mediate the relationship between interactivity and recall. In addition, we will look at whether the need for cognition and health literacy moderate the proposed mediation effects."; "In sum, we aim to answer the following research questions: through which mechanisms does interactivity affect recall of health information? Do these mechanisms differ according to individuals' level of need for cognition and health literacy?"		
METHODS		
**3a) CONSORT: Description of trial design (such as parallel, factorial) including allocation ratio**		
We describe the design of our experiment in the methods section: "A between-subjects experiment with three levels of interactivity (no interactivity, moderate interactivity, high interactivity) was conducted to investigate the effects of interactivity on cognitive involvement, perceived active control, cognitive load, and recall."		
**3b) CONSORT: Important changes to methods after trial commencement (such as eligibility criteria), with reasons**		
Not applicable.		
**3b-i) Bug fixes, Downtimes, Content Changes**		
**4a) CONSORT: Eligibility criteria for participants**		
Not applicable. We focused on the Dutch general adult population. There were no specific in- or exclusion criteria for participation in the experiment.		
**4a-i) Computer / Internet literacy**		
**4a-ii) Open vs. closed, web-based vs. face-to-face assessments:**		
"Participants were recruited from 26,000 active panel members of I&O Research, an ISO 26362–certified research bureau for access panels in market, opinion, and social research. I&O Research recruits its panel members offline (eg, from municipal registers) and self-registration is not allowed in order to prevent selection biases such as the overrepresentation of frequent internet users."		
**4a-iii) Information giving during recruitment**		
**4b) CONSORT: Settings and locations where the data were collected**		
In our paper, we did not explicitly mention "online environment" as the setting of our study, because given the fact that we examined how interactivity affects recall of website information, it is obvious that our study was conducted in an online setting.		
**4b-i) Report if outcomes were (self-)assessed through online questionnaires**		
"individuals were asked to complete a short questionnaire about health literacy, need for cognition, and educational level."; "Prior to visiting the website, individuals completed a preexposure measurement about their knowledge of the research topic and dietary supplement use."; " Once participants were finished browsing on the website, they were directed to the postmeasurement questionnaire."		
**4b-ii) Report how institutional affiliations are displayed**		
**5) CONSORT: Describe the interventions for each group with sufficient details to allow replication, including how and when they were actually administered**		
**5-i) Mention names, credential, affiliations of the developers, sponsors, and owners**		
**5-ii) Describe the history/development process**		
**5-iii) Revisions and updating**		
**5-iv) Quality assurance methods**		
**5-v) Ensure replicability by publishing the source code, and/or providing screenshots/screen-capture video, and/or providing flowcharts of the algorithms used**		
**5-vi) Digital preservation**		
**5-vii) Access**		
"On April 25, 2017, participants were invited to take part in the actual experiment."; "Then, within each stratum, participants were randomly assigned to one of the three versions of a website about vitamin B6 and dietary supplements."; "In order to make the browsing task similar to a real-life online health information search, participants had the freedom to decide what information and in which order they wanted to explore and no specific instructions or time limits were given. Participants were allowed to view the website only once."		
**5-viii) Mode of delivery, features/functionalities/components of the intervention and comparator, and the theoretical framework**		

"In interactivity research, three approaches are distinguished: structural, experiential, and message exchange. In the structural approach, interactivity is conceptualized in terms of the technical attributes of the medium. Such technical attributes include on-screen interactive features such as menus that allow user-to-system or user-to-user interactions."; "Three versions of a website were developed sharing the same content, layout, and pictures but differing in terms of levels of interactivity. In our study, ... dietary supplements."		
**5-ix) Describe use parameters**		
**5-x) Clarify the level of human involvement**		
**5-xi) Report any prompts/reminders used**		
"An invitation with the subject line "Consumer information on food and dietary supplements" was sent by email to participants on March 21, 2017."; "On April 25, 2017, participants were invited to take part in the actual experiment."		
**5-xii) Describe any co-interventions (incl. training/support)**		
No, because our study did not include any co-interventions.		
**6a) CONSORT: Completely defined pre-specified primary and secondary outcome measures, including how and when they were assessed**		
"A between-subjects experiment with three levels of interactivity (no interactivity, moderate interactivity, high interactivity) was conducted to investigate the effects of interactivity on cognitive involvement, perceived active control, cognitive load, and recall".		
**6a-i) Online questionnaires: describe if they were validated for online use and apply CHERRIES items to describe how the questionnaires were designed/deployed**		
**6a-ii) Describe whether and how "use" (including intensity of use/dosage) was defined/measured/monitored**		
**6a-iii) Describe whether, how, and when qualitative feedback from participants was obtained**		
**6b) CONSORT: Any changes to trial outcomes after the trial commenced, with reasons**		
In our paper, we did not explicitly mention "online environment" as the setting of our study, because given the fact that we examined how interactivity affects recall of website information, it is obvious that our study was conducted in an online setting.		
**7a) CONSORT: How sample size was determined**		
**7a-i) Describe whether and how expected attrition was taken into account when calculating the sample size**		
**7b) CONSORT: When applicable, explanation of any interim analyses and stopping guidelines**		
"A between-subjects experiment with three levels of interactivity (no interactivity, moderate interactivity, high interactivity) was conducted to investigate the effects of interactivity on cognitive involvement, perceived active control, cognitive load, and recall".		
**8a) CONSORT: Method used to generate the random allocation sequence**		
"Based on individuals' educational level, the sample was divided into three strata: low, medium, and high educational level."; "Then, within each stratum, participants were randomly assigned to one of the three versions of a website about vitamin B6 and dietary supplements. The random allocation was programmed by the website developer, Done Digital Kft." The method of allocation was: completely at random within each stratum".		
**8b) CONSORT: Type of randomisation; details of any restriction (such as blocking and block size)**		
"Based on individuals' educational level, the sample was divided into three strata: low, medium, and high educational level."; "Then, within each stratum, participants were randomly assigned to one of the three versions of a website about vitamin B6 and dietary supplements. The random allocation was programmed by the website developer, Done Digital Kft." The method of allocation was: completely at random within each stratum".		
**9) CONSORT: Mechanism used to implement the random allocation sequence (such as sequentially numbered containers), describing any steps taken to conceal the sequence until interventions were assigned**		
"Based on individuals' educational level, the sample was divided into three strata: low, medium, and high educational level."; "Then, within each stratum, participants were randomly assigned to one of the three versions of a website about vitamin B6 and dietary supplements. The random allocation was programmed by the website developer, Done Digital Kft." The method of allocation was: completely at random within each stratum."		
**10) CONSORT: Who generated the random allocation sequence, who enrolled participants, and who assigned participants to interventions**		
"The random allocation was programmed by the website developer, Done Digital Kft."		
**11a) CONSORT: Blinding - If done, who was blinded after assignment to interventions (for example, participants, care providers, those assessing outcomes) and how**		
**11a-i) Specify who was blinded, and who wasn't**		
Participants were blinded. They were not told beforehand that there were three versions of the website and they will see only one version.		
**11a-ii) Discuss e.g., whether participants knew which intervention was the "intervention of interest" and which one was the "comparator"**		
**11b) CONSORT: If relevant, description of the similarity of interventions**		
"In all three conditions, the information presented was identical and aimed to provide complete information about vitamin B6, its physiological effects, how it relates to food, and the risks and benefits of supplementation with vitamin B6."		
**12a) CONSORT: Statistical methods used to compare groups for primary and secondary outcomes**		
"To investigate the proposed mediations and examine whether differences exist between subgroups, the PROCESS macro for SPSS Statistics version 2.16.3 (IBM Corporation) was used [55]. PROCESS applies bootstrapping to estimate 95% bias-corrected confidence intervals for total and indirect effects. Bootstrap procedures are unaffected by violations of parametric assumptions and have higher type I error control and power than the normal theory approach [55,56].		
**12a-i) Imputation techniques to deal with attrition / missing values**		
"Due to technical issues, data of 15 respondents were lost in the preexposure measurement. Therefore, these participants were excluded from the moderated mediation analyses."		
**12b) CONSORT: Methods for additional analyses, such as subgroup analyses and adjusted analyses**		
"To examine differences between subgroups regarding the mediations (high vs low health literacy, high vs low need for cognition), model 4 (10,000 samples) was run for each group separately. Subgroups were created based on a mean split."		
RESULTS		
**13a) CONSORT: For each group, the numbers of participants who were randomly assigned, received intended treatment, and were analysed for the primary outcome**		
The exact numbers of participants per condition are stated in Figure 2 (flow diagram) in our manuscipt.		
**13b) CONSORT: For each group, losses and exclusions after randomisation, together with reasons**		
The exact number of losses and exclusions are stated in Figure 2 (flow diagram) in our manuscipt.		
**13b-i) Attrition diagram**		
**14a) CONSORT: Dates defining the periods of recruitment and follow-up**		
"An invitation with the subject line "Consumer information on food and dietary supplements" was sent by email to participants on March 21, 2017."; "On April 25, 2017, participants were invited to take part in the actual experiment."		
**14a-i) Indicate if critical "secular events" fell into the study period**		
**14b) CONSORT: Why the trial ended or was stopped (early)**		
Not applicable. The experiment did not stop or end early.		
**15) CONSORT: A table showing baseline demographic and clinical characteristics for each group**		
We did not report the sample characteristics for each group separately. Since we recruited our participants from the panel members of a research bureau who regularly take part in online (survey) research, we did not expect any difficulties that participants would encounter with browsing the website or filling out an online survey.		
**15-i) Report demographics associated with digital divide issues**		
"Slightly more female (530/983, 53.9%) than male (453/983, 46.1%) individuals participated in the study. Participants were on average aged 53.2 (SD 15.31) years, and most of them held either a medium (402/983, 40.9%) or high educational level (367/983, 37.3%; Table 2). More than half of the sample (524/983, 53.3%) had used dietary supplements in the last 12 months. Participants can be regarded as neutral toward the topic vitamin B6 since they were moderately involved (mean 3.42 [SD 1.54])."		
**16a) CONSORT: For each group, number of participants (denominator) included in each analysis and whether the analysis was by original assigned groups**		
**16-i) Report multiple "denominators" and provide definitions**		
In Figure 3 we provide information about the N per condition (how many people saw the website) and how many of them were included in the mediated moderation analysis.		
**16-ii) Primary analysis should be intent-to-treat**		
In our experiment everyone was exposed to the experimental stimulus, so the experiment took place as it was intended. There is no comparison made between users and non-users, because everyone saw one version of the website. There were no non-users.		

**17a) CONSORT: For each primary and secondary outcome, results for each group, and the estimated effect size and its precision (such as 95% confidence interval)**		
See results section.		
**17a-i) Presentation of process outcomes such as metrics of use and intensity of use**		
**17b) CONSORT: For binary outcomes, presentation of both absolute and relative effect sizes is recommended**		
Not applicable, we did not have binary outcomes.		
**18) CONSORT: Results of any other analyses performed, including subgroup analyses and adjusted analyses, distinguishing pre-specified from exploratory**		
"Manipulation Check of Interactivity: ..... Thus, the manipulation was successful."; "Descriptive Statistics of User Actions ...... Within the high interactivity condition, the proportion of participants who used all infographics was higher (36/124, 29.0%) than of those who used all dropdown menus (11/124, 8.9%)."		
**18-i) Subgroup analysis of comparing only users**		
**19) CONSORT: All important harms or unintended effects in each group**		
Not applicable. There were no harms or unintended effects in the three groups.		
**19-i) Include privacy breaches, technical problems**		
"Due to technical issues, data of 15 respondents were lost in the preexposure measurement."; "Due to personal browser settings such as a disabled JavaScript, user activity data of 524 participants out of the 983 participants were collected."		
**19-ii) Include qualitative feedback from participants or observations from staff/researchers**		
DISCUSSION		
**20) CONSORT: Trial limitations, addressing sources of potential bias, imprecision, multiplicity of analyses**		
**20-i) Typical limitations in ehealth trials**		
This is addressed in the limitations section of the discussion.		
**21) CONSORT: Generalisability (external validity, applicability) of the trial findings**		
**21-i) Generalizability to other populations**		
**21-ii) Discuss if there were elements in the RCT that would be different in a routine application setting**		
**22) CONSORT: Interpretation consistent with results, balancing benefits and harms, and considering other relevant evidence**		
**22-i) Restate study questions and summarize the answers suggested by the data, starting with primary outcomes and process outcomes (use)**		
This is described in the discussion section under principal findings.		
**22-ii) Highlight unanswered new questions, suggest future research**		
Other information		
**23) CONSORT: Registration number and name of trial registry**		
The study was not registered in a trial registration.		
**24) CONSORT: Where the full trial protocol can be accessed, if available**		
Our research protocol is archived and stored at the server of Maastricht University and can be obtained upon request from the authors. The protocol is not publicly accessible.		
**25) CONSORT: Sources of funding and other support (such as supply of drugs), role of funders**		
"This research was supported by the Netherlands Food and Consumer Product Safety Authority."		
**X26-i) Comment on ethics committee approval**		
**x26-ii) Outline informed consent procedures**		
**X26-iii) Safety and security procedures**		
**X27-i) State the relation of the study team towards the system being evaluated**		
